# Immediate Effects of Maternal Deprivation on the (Re)Activity of the HPA-Axis Differ in CD1 and C57Bl/6J Mouse Pups

**DOI:** 10.3389/fendo.2014.00190

**Published:** 2014-11-05

**Authors:** Nikolaos P. Daskalakis, Leo Enthoven, Edwige Schoonheere, Edo Ronald de Kloet, Melly S. Oitzl

**Affiliations:** ^1^Division of Medical Pharmacology, Leiden Academic Center for Drug Research, Leiden University, Leiden, Netherlands; ^2^Department of Endocrinology and Metabolism, Leiden University Medical Center, Leiden University, Leiden, Netherlands; ^3^Traumatic Stress Studies Division, Department of Psychiatry, Icahn School of Medicine at Mount Sinai, New York, NY, USA; ^4^Laboratory of Molecular Neuropsychiatry, Department of Psychiatry, Icahn School of Medicine at Mount Sinai, New York, NY, USA; ^5^PTSD Research Program, Mental Health Patient Care Center, James J. Peters Veterans Affairs Medical Center, Bronx, NY, USA; ^6^Center for Neuroscience, Swammerdam Institute for Life Sciences, University of Amsterdam, Amsterdam, Netherlands

**Keywords:** hypothalamic–pituitary–adrenal axis, corticosterone, ACTH, CRH, GR, neonate, maternal deprivation, genetic

## Abstract

The postnatal development of the mouse is characterized by a period of hypo-responsiveness of the hypothalamic–pituitary–adrenal (HPA) axis to mild stressors. Maternal deprivation (MD) during this period can disrupt the quiescence of the HPA-axis. The present study examined the influence of strain (outbred CD1 vs. inbred C57BL/6J mice) on some central and peripheral components of the HPA-axis in neonatal mice (5-day-old) in the presence of their mother or after 24 h MD (on postnatal day 4) under basal or mild stressful conditions. In the presence of the dam, adrenal corticosterone (CORT) secretion was low in both mouse strains. Compared to CD1 mice, C57BL/6J had lower CORT levels associated with higher ACTH levels and ACTH/CORT ratio (i.e., lower adrenal sensitivity to ACTH), and higher glucocorticoid receptor (GR) mRNA expression in the paraventricular nucleus. Although MD disinhibited the HPA-axis in both strains as reflected by increased basal CORT and ACTH, we found a strain-dependent pattern. MD increased CORT more in C57BL/6J compared to CD1 mice together with a lower ACTH/CORT ratio (i.e., higher adrenal sensitivity to ACTH), while GR mRNA was no longer different in the two strains. However, this increased adrenal sensitivity in maternally deprived C57BL/6J mice was not reflected in their CORT response to a subsequent novelty stressor, possibly due to an MD-induced ceiling effect in their steroidogenic capacity. In conclusion, the immediate outcome of MD depends on the genetic background of the mother–infant dyad, suggesting that maybe also the outcome in later-life cannot be generalized.

## Introduction

Maternal stimuli play a central role in the postnatal development of the hypothalamic–pituitary–adrenal axis (HPA-axis) in rodents (1, 2) especially during the stress-hyporesponsive period (SHRP). The SHRP lasts from postnatal day (pnd) 1–12 in mice, (pnd 3–14 in rats) and is characterized by low basal levels of corticosterone (CORT) and an inability to elicit a CORT response to mild stress (3, 4). Rodent dams do not leave often their nest for longer than 15–20 min during the SHRP (5). Removal of the mother for prolonged time periods (>3–8 to 24 h) has been shown to activate the HPA-axis, while the axis also becomes responsive to mild stressors, which may modulate ongoing developmental programs (6, 7).

Large individual variations in the long-term biobehavioral outcome of early-life traumatic experiences have been reported in humans (8) and rodents (9, 10). This raised the idea that early-life trauma might shape pre-existing genetic vulnerability to certain stressful conditions in later life (11). Maternal deprivation (MD) is a commonly used animal paradigm to study the consequences of early-life trauma on adult stress–responses and related behaviors (12). The MD paradigm has been applied in various designs ranging from single 24 h deprivations to repeated daily separations in time periods ranging between 3 and 8 h (9, 11).

Most of our knowledge on the effects of MD on HPA-axis and stress-related behaviors is based on research in outbred rodent strains. Although it is known that genetically selected lines of rats display differential sensitivity to the long-term effects of MD (13–15), the aspect of genetic predisposition has been given little attention. In recent experiments, we showed that responsiveness to mild stressors following prolonged maternal absence is strain-dependent (16). We actually observed that while maternally separated pups (i.e., repeatedly for 8 h) habituate toward the maternal absence *per se* by displaying low basal CORT levels (16–18), their CORT response toward a subsequent heterotypic stressor sensitizes in a strain-dependent fashion: deprived Long Evans pups were more re-active to the subsequent stressor than similarly deprived Wistar rats (16).

The inbred C57BL/6J mouse strain is most widely used as genetic background strain for engineering genetic mouse models for human diseases. A few studies compared C57BL/6J mice with common outbred mice strains (e.g., CD1 mice) on stress-related physiology and behavior. C57BL/6J and CD1 mice have differences in their circadian pattern of the stress–response (19). C57BL/6J mice have lower stress responsiveness in a light/dark exploration test for anxiety (20) and display a reduced exploration in a novel environment (21). Furthermore, CD1 mice showed better avoidance learning in a Y-maze task (22). Interestingly, C57BL/6J and CD1 mice seem to display differences on the long-term outcome of maternal separation on the stress–response, cognitive performance, anxiety/depression-like, or schizophrenia-like behaviors (23–33). Generally, the reported effects indicate more often significant effects in C57BL/6J than in CD1 mice.

Studying the immediate effects of MD on the development of the stress system responsiveness might give insights on the salient factors that influence the long-term outcome. This is an approach proven to be successful using a variety of early-life stress paradigms (18, 34). In the current study, we compared C57BL/6J with CD1 mouse pups with regard to the immediate effects of pnd 4 MD on HPA-axis stress reactivity.

## Materials and Methods

### Animals

Offspring of CD1 and C57BL/6J mice were used in this study. All mice were housed under a 12:12 h light/dark cycle (lights on at 07:00 hours) and constant temperature (23 ± 2°C) and humidity (55 ± 5%) conditions. Food (SRM-A; Hope Farms, Woerden, The Netherlands) and water (172 ml HCl/200l H_2_O) was provided *ad libitum*. Three females were mated with one male in polycarbonate boxes (820 cm^3^) containing sawdust bedding. Pregnant females were transferred to clean polycarbonate cages containing sawdust and two sheets of paper towels for nesting material. Pregnant females were checked for litters daily between 09:00 and 10:00 hours. If litters were found, the day of birth was defined as day 0 for that litter. On the day after parturition, day 1, each litter was culled to eight healthy pups (four males and four females) for the CD1 strain and to six healthy pups (three males and three females) for the C57BL/6J strain and then remained undisturbed until used in the experiment. A total of four CD1 litters and six C57BL/6J litters were used in the study. Animal experiments were approved by the Local Committee for Animal Health, Ethics, and Research of Leiden University and carried out in accordance with European Communities Council Directive 86/609/EEC.

### Experimental design

At postnatal day 4, mothers from nests randomly selected for MD (two CD1 and three C57BL/6J nests) were removed from the home cage. The home cage containing the pups was transferred to an adjacent room with similar light and temperature conditions and placed on a heating pad set at 30–33°C. Neither food nor water was available during MD. At pnd 5, half of the non-deprived (NON-DEP) and half of the deprived (DEP) pups were decapitated immediately to provide a basal sample for measurements in blood and brain. The remaining NON-DEP and DEP pups were placed individually in novel cages containing clean sawdust bedding on heating pads set at 30–33°C and decapitated after 30 min to provide a novelty stress sample.

### Blood processing and hormone determination

Trunk blood from all decapitated pups was collected in EDTA-coated microcentrifuge tubes; plasma was extracted and stored frozen at −20°C until hormone determination. ACTH was measured by radioimmunoassay (RIA; MP Biomedicals, LLC, NY, USA; sensitivity 10 pg/ml, intra-assay variation 4.1%, inter-assay variation 4.4%) as described before (16). CORT was measured by RIA (MP Biomedicals, LLC, NY, USA; sensitivity 1.25 ng/ml, intra-assay variation, 4.4%, interassay variation 6.5%) as described before (16). We calculated the ratio of ACTH to CORT as an indirect measure of adrenal sensitivity to ACTH (18).

### *In situ* hybridization

Frozen brains and pituitaries were sectioned at −20°C in a cryostat microtome at 16 μm in the coronal plane. Sections were thaw-mounted on poly-l-lysine coated slides, air-dried, and kept at −80°C. *In situ* hybridization using 35S-UTP labeled ribonucleotide probes [CRH and glucocorticoid receptor (GR)] was performed as described previously (17, 18). The slides were opposed to Kodak Biomax MR film (Eastman Kodak Co., Rochester, NY, USA) and developed. Autoradiographs were digitized, and relative expression of CRH and GR mRNA was determined by computer-assisted optical densitometry (analysis 3.1, Soft Imaging System GmbH, Münster, Germany). The mean of four to six measurements was calculated for each mouse.

### Statistics

Data were analyzed by analysis of variance (ANOVA) using strain (CD1 or C57BL/6J), treatment (NON-DEP or DEP), and time (basal or novelty) as fixed factors. When appropriate, *post hoc* Tukey test was performed. The initial analysis included sex as a factor; once it was determined that sex was not a significant factor, the data were collapsed across this variable. The level of significance was set at *P* < 0.05. Data are presented as mean ± SEM.

## Results

### Weight

Two-way ANOVA revealed main effects of strain (*F*_1,64_ = 141.34; *p* < 0.001) and treatment (*F*_1,64_ = 141.34; *p* < 0.001). C57BL/6J were lighter than CD1 mice (*p* < 0.001) in both treatment conditions (Table 1). DEP pups were lighter than NON-DEP pups (*p* < 0.001 for both strains).

**Table 1:** **Body weight (grams) of non-deprived (NON-DEP) and 24 h deprived (DEP) pups in CD1 and C57BL/6J mice at postnatal day 5**.

Strain	NON-DEP			DEP			% Change
	Mean	*N*	SEM	Mean	*N*	SEM	
CD1	3.15	16	0.06	2.51^#^	16	0.08	↓20
C57BL/6J	2.51^$^	16	0.10	1.78^#$^	17	0.11	↓29

*Data represent mean ± SEM. # vs. NON-DEP, $ vs. CD1*.

*Significance level was set at *p* = 0.05*.

### ACTH

**Figure 1:**
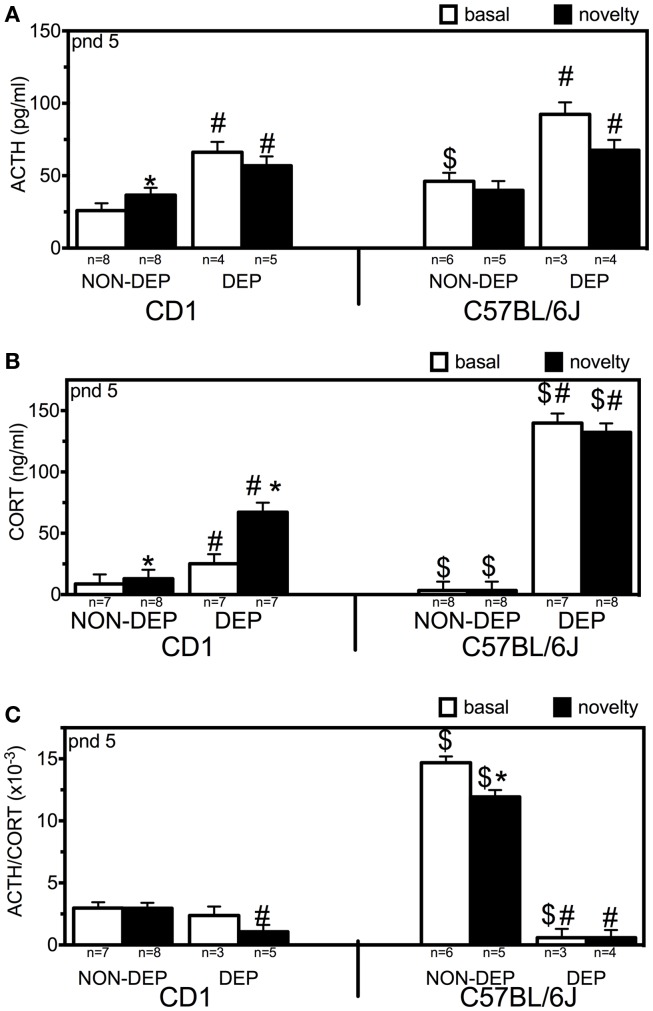
**ACTH [(A); picogram/milliliter], corticosterone [(B); CORT in nanogram/milliliter] blood plasma levels, and their ratio [(C); ACTH/CORT] of non-deprived (NON-DEP) and 24 h deprived (DEP) pups measured at basal conditions (basal; white bars) or after 30 min of novelty exposure (novelty; black bars) at postnatal day (pnd) 5**. Data represent mean ± SEM. * vs. basal, # vs. NON-DEP, $ vs. CD1. Significance level was set at *p* = 0.05.

Three-way ANOVA revealed main effects of strain (*F*_1,42_ = 10.79; *p* < 0.001), treatment (*F*_1,42_ = 53.65; *p* < 0.001), and interaction of treatment and time (*F*_1,42_ = 4.41; *p* = 0.043) (Figure 1A). Strain differences were found at NON-DEP basal levels (*p* = 0.005). Novelty exposure increased ACTH levels in CD1 (↑41%, *p* = 0.025) but not in C57BL/6J mice. After 24 h MD, ACTH basal levels were elevated (↑156%, *p* = 0.001 for CD1; ↑100%, *p* = 0.006 for C57BL/6J). Subsequent novelty exposure did not produce further increase in ACTH in either CD1 or C57BL/6J mice, while in both strains ACTH levels were higher than the respective NON-DEP levels (*p* = 0.010 for CD1, *p* = 0.040 for C57BL/6J).

### Corticosterone

Three-way ANOVA revealed main effects of strain (*F*_1,59_ = 59.86; *p* < 0.001), treatment (*F*_1,59_ = 248.76; *p* < 0.001), interaction strain and treatment (*F*_1,59_ = 83.52; *p* < 0.001), interaction strain and time (*F*_1,59_ = 6.38; *p* = 0.015), and interaction of strain, treatment, and time (*F*_1,59_ = 4.50; *p* = 0.039) (Figure 1B). Novelty exposure increased CORT levels in CD1 (↑50%, *p* = 0.002) but not in C57BL/6J mice. After 24 h MD, CORT basal levels were elevated in both strains (↑191%, *p* < 0.001 for CD1; ↑4099%, *p* < 0.001 for C57BL/6J). Subsequent novelty exposure further increased CORT only in CD1 mice (additional ↑167%, *p* < 0.001), while in both strains CORT levels were higher than the respective NON-DEP levels (*p* < 0.001). Strain differences were found at all four conditions: C57BL/6J CORT levels being lower than in CD1 at NON-DEP conditions (*p* < 0.001 for both basal and novelty), and higher at DEP conditions (for basal: *p* < 0.001, for novelty: *p* = 0.003).

### ACTH/CORT ratio

Three-way ANOVA revealed main effects of strain (*F*_1,40_ = 126.05; *p* < 0.001), treatment (*F*_1,40_ = 290.46; *p* < 0.001), time (*F*_1,40_ = 6.24; *p* = 0.018), interaction strain and treatment (*F*_1,40_ = 196.03; *p* < 0.001), and interaction of strain, treatment, and time (*F*_1,40_ = 6.12; *p* = 0.019) (Figure 1C). At NON-DEP basal conditions, C57BL/6J displayed much higher ACTH/CORT than CD1 mice (↑393%, *p* < 0.001). Novelty exposure decreased the ratio in C57BL/6J (↓19%, *p* = 0.048) but not in CD1 mice. After 24 h MD, ACTH/CORT ratio decreased in C57BL/6J (↓96%, *p* < 0.001) but not in CD1 mice in such an extent that the C57BL/6J displayed even less ratio than CD1 mice (*p* = 0.029). For both strains, ACTH/CORT ratios after subsequent novelty exposure were lower than the respective NON-DEP levels (CD1: *p* = 0.004, C57BL/6J: *p* < 0.001).

### CRH mRNA expression in the PVN

**Figure 2:**
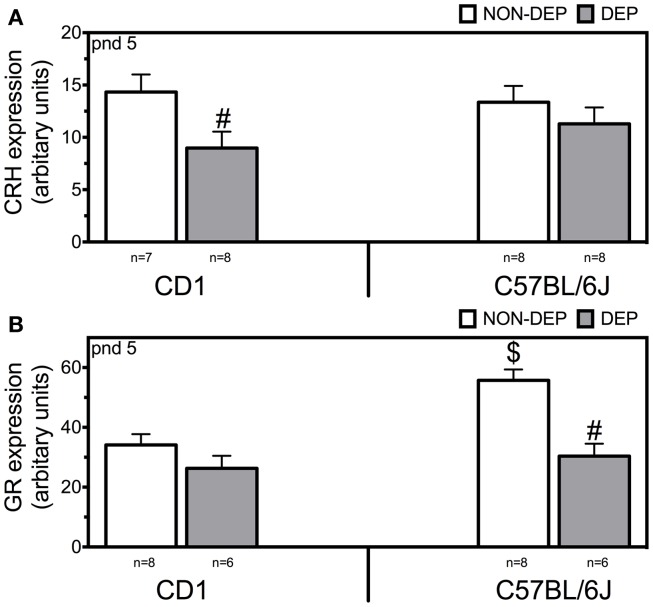
**CRH (A) and GR (B) mRNA expression in the paraventricular nucleus of the hypothalamus measured in non-deprived (NON-DEP) and deprived (DEP) mice at postnatal day (pnd) 5**. Data represent mean ± SEM. # vs. NON-DEP, $ vs. CD1. Significance level was set at*p* = 0.05.

Two-way ANOVA revealed main effects of treatment (*F*_1,30_ = 5.41; *p* = 0.028) (Figure 2A). Twenty-four hours of MD downregulated CRH mRNA (*p* = 0.036) in CD1 mice but not in C57BL/6J.

### GR mRNA expression in the PVN

Two-way ANOVA revealed main effects strain (*F*_1,27_ = 10.77; *p* = 0.003), treatment (*F*_1,27_ = 17.97; *p* < 0.001), and interaction of strain and treatment (*F*_1,27_ = 5.02; *p* = 0.035) (Figure 2B). At basal conditions, C57BL/6J displayed higher levels of GR mRNA than CD1 mice (*p* = 0.002). Twenty-four hours of MD downregulated GR mRNA in C57BL/6J (*p* < 0.001).

### GR mRNA expression in pituitary (data not shown)

There were no main effects of strain or treatment on GR mRNA in pituitary.

## Discussion

Our data show that the two mouse strains, CD1 and C57BL/6J mice, differ in the neonatal HPA-axis activity at basal conditions as well as after a 24 h MD period.

Regarding basal HPA-axis activity, C57BL/6J displayed higher ACTH and lower CORT than CD1 mice, indicating lower basal adrenal sensitivity to ACTH as reflected by a higher ACTH/CORT ratio. Additionally, basal GR mRNA expression in the PVN is higher than in CD1 mice. We propose that this increased GR mRNA expression might be a result of the lower CORT production. The higher GR mRNA is not likely to be an indication of stronger negative feedback capacity because there was no strain difference in basal CRH mRNA expression in the PVN or GR mRNA expression in the pituitary. Exposure of NON-DEP pups to novelty resulted in a subtle statistically significant rise in both ACTH and CORT in CD1 mice only. This finding underlines strain-dependent effects and confirms that the SHRP is a period of stress-hypo-responsiveness (3).

Maternal deprivation elicited in both strains the expected increase of ACTH (35) and CORT (35, 36). ACTH rose at a similar extent in both strains. CORT levels were dramatically increased in C57BL/6J compared to a more moderate increase in CD1 pups. Previous findings in rats showed that during the time-course of 24 h maternal separation, adrenal sensitivity to stress increased (37) through increases in melanocortin type 2 receptors for ACTH (16) or other mechanisms (38) in a strain-dependent manner (16). The decrease in ACTH/CORT ratio in C57BL/6J compared to CD1 pups (from higher ACTH/lower CORT to comparable ACTH/higher CORT) indicates that, C57BL/6J after MD are no longer less sensitive to ACTH than CD1 mice at the adrenal level, but actually they display increased adrenal sensitivity compared to CD1 mice. In that, CORT secretion may be influenced also by factors other than ACTH, direct measures of neonatal adrenal sensitivity to ACTH need to be undertaken in future experiments. Only CD1 mice displayed a CORT response to novelty stress after MD. The absence of an additional novelty-induced CORT increase in C57BL/6J might be related to a ceiling effect in their steroidogenic capacity.

It is interesting that C57BL/6J do not show the expected reduction in CRH mRNA expression following MD (35, 39) that was seen in the CD1 pups. This might be associated with the reduction in GR mRNA expression in the PVN and, thus, with potentially less efficient negative feedback actions of CORT at the cells that produce and release CRH. This might be an indication that, in C57BL/6J, MD causes a greater disruption of SHRP, which is characterized by enhanced negative feedback (40).

Another contributing factor to the strain differences here might be the transcortin levels and ultimately the free (biologically active) CORT, which is the HPA-axis feedback signal. RIA does not distinguish between free and transcortin-bound cortisol. Transcortin levels are low during SHRP (41) and strain differences are possible. Peripheral and central metabolic factors (e.g., blood glucose, arcuate nucleus NPY) can also mediate the activation of the HPA-axis induced by maternal separation (42, 43). Indeed, in terms of body weight changes, MD caused the greatest metabolic challenge in C57BL/6J pups, which also displayed the highest activation of the HPA-axis expressed by CORT. Other factors not related to feeding might be also involved. Actually feeding is more related with the adrenal CORT secretion and tactile stimulation more related to pituitary ACTH release (1).

We have to acknowledge some limitations of the study. Pre-weaning pups from small litters (<5 pups) have higher body weight and higher basal CORT levels than pups from large litters (>15 pups) (44). The C57BL/6J litters are naturally smaller in size than the CD1 litters. This has created an unavoidable, without cross-fostering, confound that might have interfered with the strain differences reported. We opted for an equal sex-ratio (1:1) that removed the sex-ratio bias. Nevertheless, the litter-size difference between the strains was small (two pups) and did not seem to have a noticeable effect; in this experiment, the pups of the C57BL/6J strain (with the smaller litter size of six pups) displayed lower body weight and lower basal CORT than the pups of the CD1 strain (with the larger litter size of eight pups). Future studies could illuminate the role of litter-size, but also of the basal mother–pup interactions and other related epigenetically mediated mechanisms (45) on the neonatal basal and post-MD HPA-axis activity.

Specific genetic contributions could be clarified in the future with the use of transgenic mice, but the strain differences in immediate effects of MD observed, here, in mice and, previously, in rats (16) emphasize the importance of genetic background on the effects of early maternal environment on the development of the stress system. Late-life consequences may also depend on genetic background, but this remains to be tested.

## Conflict of Interest Statement

The authors declare that the research was conducted in the absence of any commercial or financial relationships that could be construed as a potential conflict of interest.
